# Simultaneous treatment of renal cysts and ipsilateral stones with retroperitoneoscopic surgery: report of two cases

**DOI:** 10.1016/j.ijscr.2025.111504

**Published:** 2025-06-13

**Authors:** Minh Tuan Nguyen, Huu Thanh Nguyen, Minh Phuc Cao, Hong Thai Nguyen, Ha Chau Trinh, Truong Giang Nguyen

**Affiliations:** a Department of Urological Surgery, Bach Mai Hospital, Hanoi, Viet Nam; b Department of Surgery, Hanoi Medical University, Hanoi, Viet Nam; c Radiology Center, Bach Mai Hospital, Hanoi, Viet Nam

**Keywords:** Retroperitoneoscopy, Cyst drainage, Renal cysts, Renal calculis

## Abstract

**Introduction and importance:**

A simple kidney cyst is the most common type of renal cyst. These cysts are usually asymptomatic and detected incidentally upon radiological exams of the abdomen. Currently, there are many methods of treating renal cysts, in which retroperitoneoscopy to remove the cyst is a safe and more effective method. However, the simple renal cyst with renal pelvis stones will raise a question in the approach.

**Case presentation:**

In this report, we present two cases, admitted to the hospital with abdominal pain and diagnosed with a simple renal cyst with renal calculis. The two patients underwent retroperitoneal laparoscopic treatment of renal cyst and renal calculi in one surgery.

**Clinical discussion:**

We want to discuss and share our experience in surgically treating these exceptional cases.

**Conclusion:**

In two cases, we chose the retroperitoneoscopic technique to remove the renal cyst and the renal stone, which resulted in good treatment results. This is an effective and safe alternative for the simultaneous treatment of renal cysts and ipsilateral renal calculi in carefully selected patients.

## Highlights

• Simple renal cysts and renal calculi are two common urinary system diseases with many effective treatment methods.
• However, the co-occurrence of both simple renal cysts and renal pelvis stones in the same kidney may result in different intervention strategies, depending on each patient's case.
• In this case, we chose the retroperitoneal endoscopic technique to resect the renal cyst tip and remove the renal pelvis stones, which has brought about good treatment results.
• This is an effective and safe alternative for simultaneously treating renal cysts and calculi on the same side in carefully selected patients.

## 1 Background

Simple renal cysts and renal calculi are two common groups of diseases in kidney diseases. Simple renal cysts often appear alone in each kidney, but in some cases, multiple cysts occur in the same kidney. Simple renal cysts are classified according to the Bosniak classification 2019 with four different levels based on imaging characteristics of contrast-enhanced CT, helping to predict the risk of malignancy and suggesting monitoring or treatment [1]. Simple cysts usually do not interfere with kidney function and do not typically affect renal function tests. However, in some cases, if not treated promptly, simple cysts can lead to impaired renal function. Most people are not aware of having them. Rarely a simple cyst can rupture and bleed, become infected, or grow so large that it causes a mass effect on other organs and abdominal pain and discomfort. Treatment methods for renal cysts include (1) aspiration and sclerotherapy and (2) laparoscopic surgery to remove the cyst. Laparoscopic decortication is indicated for Bosniak classification type I renal cysts and is a safe and effective method with a high success rate [2,3].

Renal calculi are the most common urinary disease in the kidney. Patients often come to the hospital with symptoms of low back pain, sometimes discovered by chance during examination. It can cause serious complications if not treated and handled promptly. Currently, there are many methods of treating this disease, such as percutaneous nephrolithotomy, flexible ureteroscopy with holmium laser, retroperitoneoscopic or open surgery to remove stones. Of these, percutaneous nephrolithotomy and flexible ureteroscopy with holmium laser are becoming more popular and can access most types of renal calculi with high and safe stone-free treatment efficiency [4,5].

The two diseases of renal cysts and renal calculi currently have many different treatment methods with apparent effectiveness. However, the simultaneous appearance of renal cysts and calculi in the same patient can make the treatment method somewhat different. This case may not be rare, but not many articles or documents mention it. Therefore, our research team would like to report a clinical case of a patient with a combination of both renal cysts and renal calculi with the hope of sharing our experience and receiving positive feedback from our colleagues. The report focuses on the surgical approach and method. This case report has been reported in line with the SCARE checklist [6].

## 2 Case presentation

### 2.1 Case 1

A 54-year-old female patient with no relevant medical or family history visited our hospital with chief complaints of lower back pain for 1 week, ASA I, BMI 24.4. Her vital signs were within normal limits. Physical examinations were within normal limits. Laboratory examinations found no abnormalities, including hematology, renal function, and urinalysis.

**Fig. 1:**
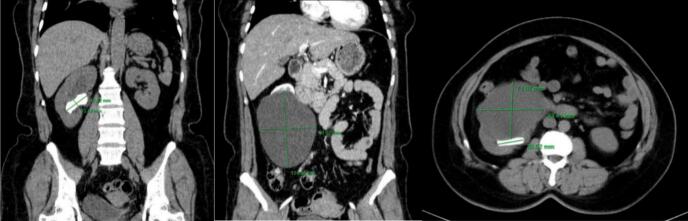
Image of the right simple renal cyst and ipsilateral renal calculi on CT of patient 1.

Computed tomography (CT) was performed and showed the size of the right kidney normal. The renal parenchyma had a cystic lesion, reduced density at the lower pole of the kidney, thin wall, homogeneous fluid density in the cyst, no contrast enhancement in the arterial phase, and contains no calcifications, size 124 × 72 × 83 mm, causing compression and deformation of the remaining renal parenchyma, Bosniak I classification (Fig. 1). The renal pelvis was slightly dilated, with a stone measuring 42 × 13 mm. The ureter was not dilated. The renal cyst and renal calculi were separated by a thin layer of renal parenchyma, with no major blood vessels passing through when observed on contrast-enhanced CT scan. The patient was then indicated for retroperitoneoscopic surgery to remove the right renal cyst and ipsilateral renal calculi. We used the balloon dilation technique to develop the retroperitoneal space due to its advantages. We addressed the renal cyst first, as it served as the initial gateway. After excising the cyst wall, accessing the renal pelvis to remove the stones became much easier. After exposing the cyst wall, we decided to decompress the cyst by aspirating its fluid to create sufficient working space before excising it.

The patient was placed in the left lateral decubitus position at 90^o^, with padding placed under the opposite lumbar rib and the operating table at a moderate angle. We used four trocars with the following positions: the first trocar (10 mm) was placed in the mid-axillary line about 2 cm below the XII rib; the second trocar (5 mm) was placed in the posterior axillary line at the angle of the XII rib with the back muscle mass; the third trocar (10 mm) was placed in the posterior axillary line, about 5 cm below the second trocar; the fourth trocar (5 mm) was placed in the anterior axillary line, 4 cm above the anterior iliac spine. The operation time was 124 min, with about 300 ml of serous fluid aspirated from the cyst and about 50 ml of blood loss during surgery.

The patient's postoperative course was uneventful, and no complications were observed. The patient was discharged 5 days after the operation. After 4 weeks of follow-up, her pain disappeared, the abdominal ultrasound showed no signs of fluid collection in the right retroperitoneal, the JJ catheter was in a normal position, and there was no increased echo image with acoustic shadowing in the renal pelvis. The pathological results were benign. The patient had the JJ catheter removed afterward. During the process of removing stones from the renal pelvis, we used Kelly forceps. The impact between the Kelly forceps and the stone surface posed a risk of leaving behind small stone fragments. Additionally, extensive dissection of the retroperitoneal space could irritate the ureter, causing postoperative spasms, which could increase renal pelvic pressure and raise the risk of urine leakage through the renal pelvis incision site. Therefore, we placed a JJ stent to help eliminate residual stone fragments and reduce pressure on the renal pelvis incision site.

These were among our first cases, so we decided to keep the patients hospitalized for 5 days to ensure close monitoring and to promptly detect any potential complications such as bleeding or urine leakage. Clinically, on the third postoperative day, after removing the urinary catheter, we observed no further output from the renal drain, and it could be safely removed. The patients could have been discharged the following day.

### 2.2 Case 2

A 78-year-old male patient with no relevant medical or family history, was admitted to the hospital with right lower back pain for 2 weeks, ASA I, BMI 20.76. His vital signs were within normal limits. Physical examinations were within normal limits. Laboratory examinations including hematology, renal function, and urinalysis found no abnormalities.

A renal CT scan was performed and showed that the right kidney was of normal size. The renal parenchyma had several cystic lesions, reduced density at the lower pole of the kidney, thin walls, homogeneous fluid density in the cyst, no contrast enhancement in the arterial phase, no calcifications, causing compression and deformation of the remaining renal parenchyma, Bosniak I classification, the largest cyst was 70 mm in diameter. The renal pelvis was slightly dilated, with several small stones, the largest stone was 16 mm in size. The ureter was not dilated. Renal cysts and kidney stones are separated by a thin layer of renal parenchyma, with no large blood vessels passing through when observed on contrast-enhanced CT scans.

The patient was then indicated for retroperitoneoscopic surgery to remove the right renal cyst and ipsilateral renal calculi. The patient position and location of the trocars were the same as in the first case. The surgery lasted about 110 min, with about 200 ml of cyst fluid aspirated from the cyst and about 30 ml of blood loss during surgery.

The patient's postoperative course was uneventful, and no complications were observed. The patient was discharged 5 days after the operation. After 4 weeks of follow-up, his pain disappeared, the abdominal ultrasound showed no signs of fluid collection in the right renal fossa, the JJ catheter was in a normal position, and there was no increased echo image with acoustic shadowing in the renal pelvis—the pathological results of the benign renal cyst. The patient had the JJ catheter removed afterward.

## 3 Discussion

A simple kidney cyst is a pocket of fluid that originates from the surface of the kidney and is contained by a thin wall [7]. This is an acquired disease with an unknown cause, but age and male sex are the leading risk factors [8]. Simple renal cysts are often asymptomatic and are discovered incidentally during ultrasound or CT scans. They are characterized by an absence of echoes, no septations, and has no central or peripheral calcification, and a thin wall [9].

In 2014, Chen et al. reported 15 well-selected patients who successfully underwent percutaneous nephrolithotripsy and simultaneous intrarenal cyst marsupialization; during a median follow-up period of 21 months, all patients showed calculi clearance and noticeable cyst regression, and no severe intraoperative or postoperative complications were noted [10]. Yang et al. (2018) reported a clinical case wherein a peripelvic renal cyst combined with renal calculi was successfully treated with percutaneous nephroscopy [11]. Although effective, percutaneous nephroscopy is suitable for a limited number of cases in which patients exhibit a solitary posterior or parapelvic medium- to large-sized renal cyst accompanied by ipsilateral calculi. Moreover, the risk of uncontrolled bleeding requiring angioembolization should be seriously considered, especially for patients with solitary kidneys. In 2019, Zhu Zewu et al. used flexible ureteroscopy with holmium laser to simultaneously treat simple renal cysts and ipsilateral renal calculis with stones <2.5 cm in size [12]. Although this technique is minimally invasive, it can be seen that it is suitable for small renal calculi because large stones prolong the lithotripsy time and increase the risk of complications due to water injection, as well as the inability to obtain a pathological sample during treatment.

In the first case, there was a single renal cyst but the renal pelvis stone was 42 × 13 mm in size, which was not suitable for flexible ureteroscopy lithotripsy (Fig. 1). In the second case. However, the renal pelvis stone was small, and there were many renal cysts inside, so it was difficult to lithotripsy with a flexible ureteroscopy and open the renal cyst (Fig. 6). After reviewing the general condition of the patients as well as the imaging, we decided to choose the retroperitoneoscopic method of renal cyst resection for the two patients. For renal pelvis stones, we offer two intervention options for the patient. The first option is to remove the renal cyst and remove the renal pelvis stone at the same time if the position of the renal pelvis stone after removing the cyst is favorable. The second option is to remove the renal cyst and consider percutaneous nephrolithotomy in another intervention later.

**Fig. 2:**
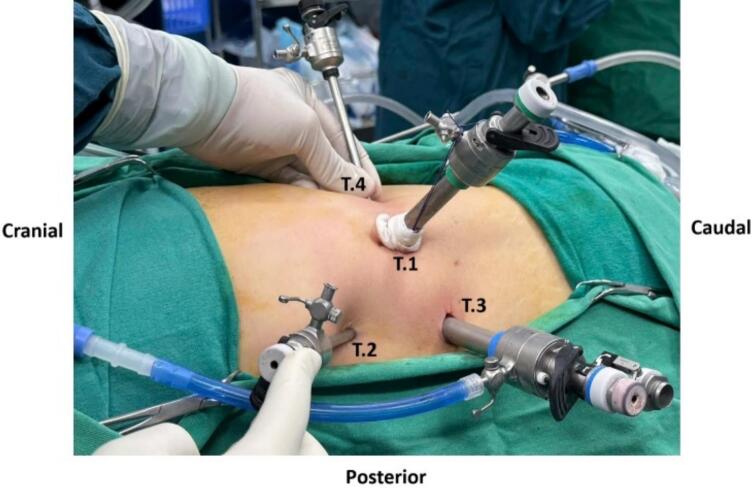
Trocar placement.

**Fig. 3:**
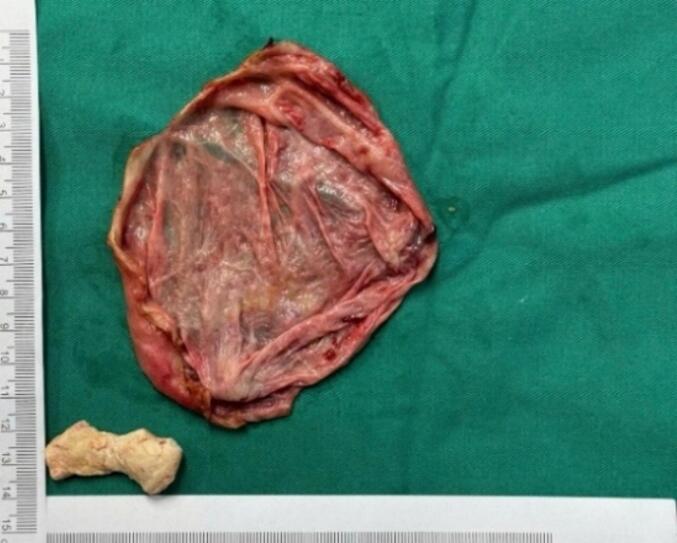
Postoperative specimen.

**Fig. 4:**
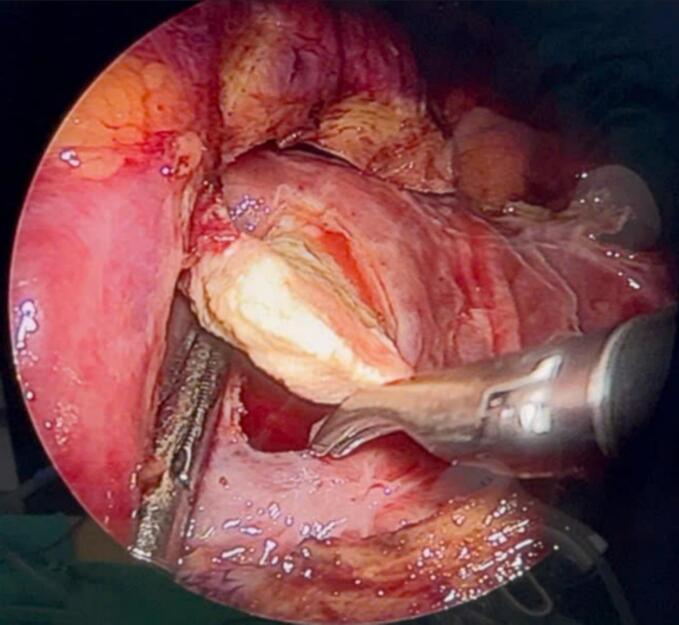
Opening the renal pelvis to remove stones.

**Fig. 5:**
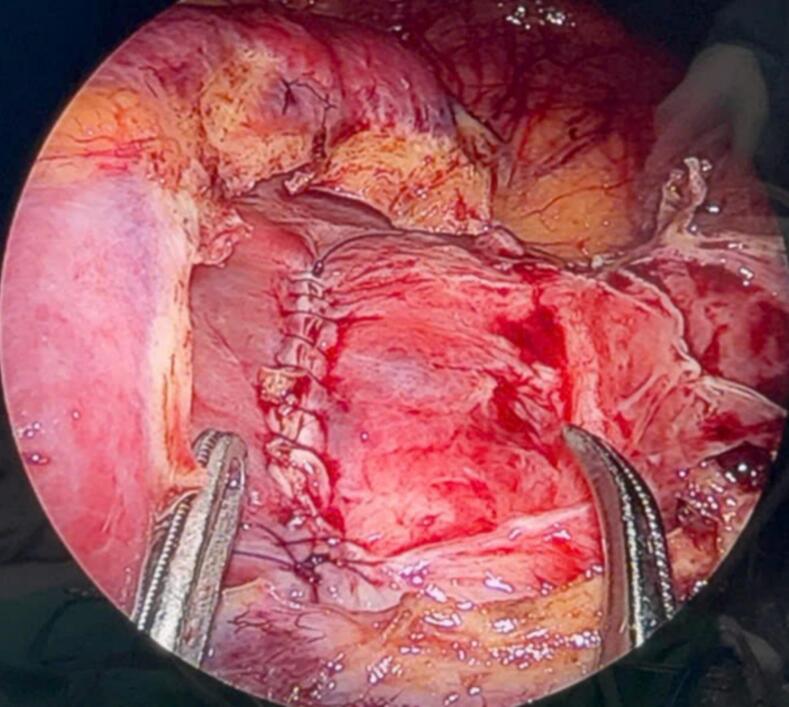
Image of kidney parenchyma sutured after kidney stone removal.

During the surgery, the renal cyst removal step went smoothly. When observing and exploring the renal parenchyma after removing the cyst, we felt the stone and moved it with a grasper and hook, but it would be better if there were an intraoperative ultrasound in similar cases. Because the remaining part of the renal cyst is thin and has no large blood vessels, we decided to open the renal pelvis to remove the stone (Fig. 4, Fig. 5, Fig. 7, Fig. 8). Then, we placed a JJ ureteral stent and sutured the incision site. The patient had a retroperitoneal drainage and a urinary catheter placed. Simultaneous renal cysts and ipsilateral renal calculi treatment may reduce recovery time and medical costs. Therefore, surgeons seek to treat both conditions simultaneously, although the coexistence of both conditions on the same side is uncommon (Fig. 2, Fig. 3).

**Fig. 6:**
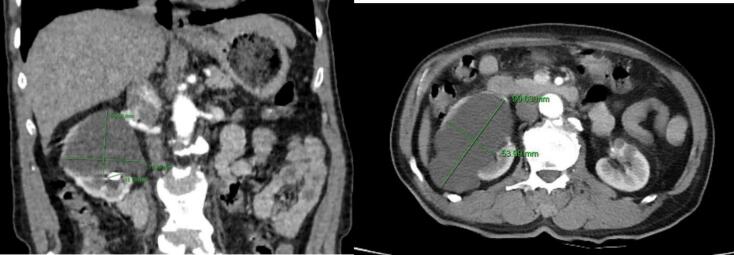
Image of the right renal cyst and renal calculi on CT scan of patient 2.

**Fig. 7:**
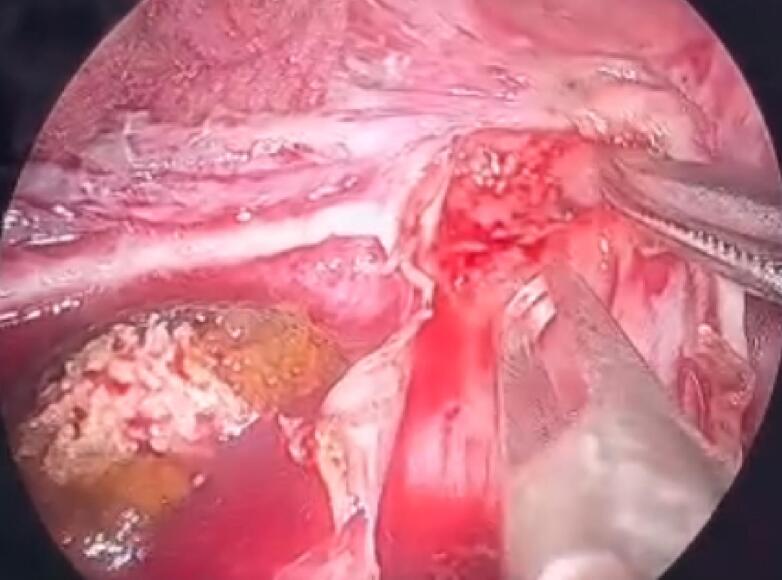
Opening the renal pelvis to remove stones.

**Fig. 8:**
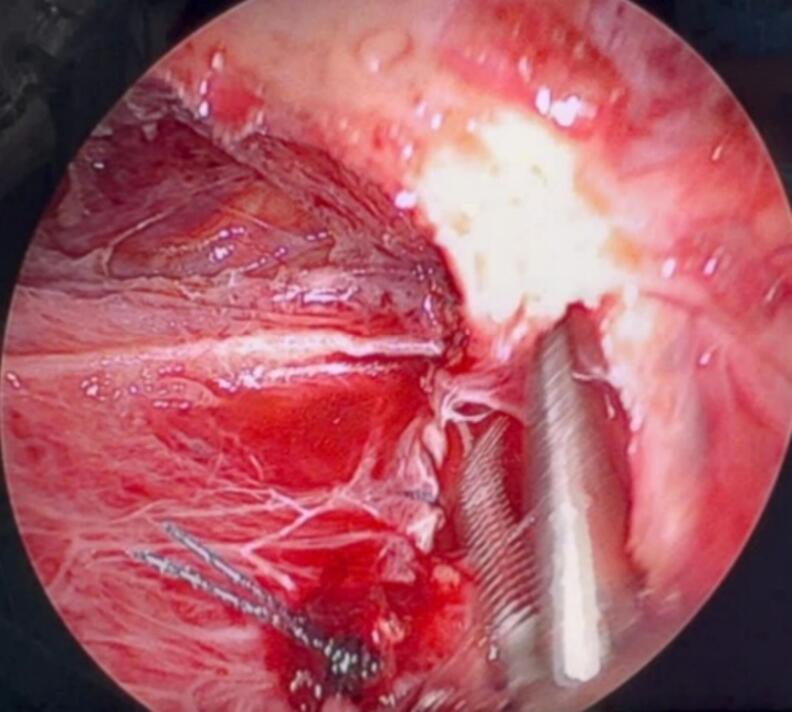
Image of kidney parenchyma sutured after kidney stone removal.

The most significant advantage of this technique is its ability to treat renal stones and cysts in a single surgery. Additionally, we can thoroughly manage renal cysts by completely excising the cyst wall (compared to simple cyst aspiration) and reduce the risk of postoperative bleeding, as the entire surgical field is carefully inspected before trocar removal (compared to percutaneous nephrolithotomy). The limitation of this technique is that in cases of staghorn stones, deeply located stones, or stones scattered across multiple locations far from the renal cyst, accessing and completely removing all stones may become more challenging. Therefore, this method is most suitable for large, simple renal cysts accompanied by renal pelvic stones that can be removed entirely through the renal pelvis incision. It is also ideal for patients with stones and cysts close to each other, allowing for convenient intervention in a single surgery.

## 4 Conclusion

In two cases, we chose the retroperitoneoscopic technique to remove the renal cyst and the renal stone, which resulted in good treatment results. This is an effective and safe alternative for the simultaneous treatment of renal cysts and ipsilateral renal calculi in carefully selected patients.

## CRediT authorship contribution statement

**Minh Tuan Nguyen:** Conceptualization, Visualization, Writing – original draft. **Huu Thanh Nguyen**: Writing – review & editing. **Minh Phuc Cao:** Writing – review & editing. **Hong Thai Nguyen:** Resources. **Ha Chau Trinh:** Resources **. Truong Giang Nguyen:** Conceptualization, Visualization, Writing – original draft.

## Consent for publication

This case report has gotten approval for publication from ethics of local IRB.

## Ethics approval

This case report has gotten approval from ethics committee of Bach Mai Hospital (01/QĐ-IRB, dated on 10, Januaary, 2025).

## Guarantor

Minh Tuan Nguyen, MD, PhD

Truong Giang Nguyen, MD, PhD

## Research registration number

This study does not require registration.

## Funding

No funding involved in this case report.

## Declaration of competing interest

The authors declare that they have no competing interests.

## Data Availability

No datasets were generated or analysed during the current study.
